# Plasma-activated medium induces ferroptosis by depleting FSP1 in human lung cancer cells

**DOI:** 10.1038/s41419-022-04660-9

**Published:** 2022-03-07

**Authors:** Ara Jo, Jin Hee Bae, Yu Jeong Yoon, Tae Hun Chung, Eun-Woo Lee, Young-Ho Kim, Hea Min Joh, Jin Woong Chung

**Affiliations:** 1 Department of Biological Sciences, Dong-A University, Busan, 49315 Korea; 2 Department of Molecular Biology and Immunology, College of Medicine, Kosin University, Busan, Korea; 3 Department of Materials Physics, Dong-A University, Busan, 49315 Korea; 4 Metabolic Regulation Research Center, Korea Research Institute of Bioscience and Biotechnology (KRIBB), Daejeon, 34141 Korea; 5 Department of Functional Genomics, University of Science and Technology (UST), Daejeon, 34141 Korea

**Keywords:** Non-small-cell lung cancer, Drug development

## Abstract

Cold atmospheric plasma (CAP) that generates reactive oxygen species (ROS) has received considerable scientific attentions as a new type of anticancer. In particular, an indirect treatment method of inducing cancer cell death through plasma-activated medium (PAM), rather than direct plasma treatment has been well established. Although various cell death pathways such as apoptosis, necroptosis, and autophagy have been suggested to be involved in PAM-induced cell death, the involvement of ferroptosis, another type of cell death regulated by lipid ROS is largely unknown. This study reports, that PAM promotes cell death via ferroptosis in human lung cancer cells, and PAM increases intracellular and lipid ROS, thereby resulting in mitochondrial dysfunction. The treatment of cells with N-acetylcysteine, an ROS scavenging agent, or ferrostatin-1, a ferroptosis inhibitor, protects cells against PAM-induced cell death. Interestingly, ferroptosis suppressor protein 1 (FSP1) is downregulated upon PAM treatment. Furthermore, the treatment of cells with iFSP1, an inhibitor of FSP1, further enhances PAM-induced ferroptosis. Finally, this study demonstrates that PAM inhibits tumor growth in a xenograft model with an increase in 4-hydroxynoneal and PTGS2, a byproduct of lipid peroxidation, and a decrease in FSP1 expression. This study will provide new insights into the underlying mechanism and therapeutic strategies of PAM-mediated cancer treatment.

## Introduction

Cold atmospheric plasma (CAP), an ionized gas generated at room temperature, contains neutral molecules and atoms, electrons, various types of ions, excited species, and radicals [1–3]. Among them, reactive oxygen species (ROS) are considered significant factors that can lead to cancer cell death. Since CAP is a convenient and abundant source of ROS, CAP has been applied to cancer treatment in vitro and in vivo [4–8]. There are two methods for treating cancer cells with CAP. One is to induce cancer cell death by directly irradiating plasma, which has been studied since the early stages of plasma medicine [9–14]. The other is an indirect treatment method that has recently been studied [15–19]. Even now, studies to explore the mechanism underlying cancer cell death by direct and indirect treatment of CAP have been actively conducted [20–24].

In our previous study, it was found that directly treated oxygen-containing CAP can induce DNA damage and cell death in human lung cancer cells [25]. In addition, we reported that cancer cell death is induced through indirect treatment using a plasma-activated medium (PAM), and PAM increases the intracellular ROS levels and regulates JNK/p53/Bax signaling, resulting in apoptosis [26–28]. Other studies have also reported that PAM causes necroptosis and autophagy, including apoptosis [29, 30]. However, to our knowledge in-depth studies on the mechanism of PAM-induced cancer cell death are scarce.

Among the ROS-associated cell signaling pathways, ferroptosis has been associated with PAM. Ferroptosis is newly recognized as a form of regulated cell death that is distinct from apoptosis, necroptosis, and autophagic cell death [31]. It is can occur via increased lipid peroxidation and iron accumulation [32, 33]. Glutathione peroxidase 4 (GPX4) reduces lipid peroxide to lipid alcohol using glutathione (GSH), thereby protects cells from ferroptosis [34, 35]. Therefore, the inhibition of GPX4 or depletion of GSH can induce ferroptosis in various cell types. Nuclear factor erythroid 2-related factor 2 (NRF2) is a master transcription factor for the antioxidant defense mechanism [36, 37]. NRF2 is also a potent ferroptosis suppressor protein (FSP) that determines ferroptosis sensitivity [38]. Recently, apoptosis-inducing factor mitochondria-related 2 (AIFM2) has been reported to act as antiferroptotic protein that was not recognized previously and was then renamed FSP1 [39, 40]. FSP1/AIFM2 protects cells from ferroptosis by reducing ubiquinone, an oxidized form of coenzyme Q10, thereby promoting the recycling of coenzyme Q10 at the plasma membrane, independently of GPX4.

Given that PAM generates ROS to induce cell death, we hypothesized that ferroptosis might also be involved in PAM-mediated cell death. In support of this hypothesis, we observed several characteristics of ferroptosis in PAM-induced cell death. Here, we showed that PAM leads to changes in the levels of major proteins involved in ferroptosis and the accumulation of labile iron and lipid peroxidation, thus, meeting the principal criterion for ferroptosis. Moreover, we confirmed that PAM regulates NRF2/FSP1 expression. PAM showed cancer-inhibiting ability in vivo and regulated ferroptosis without causing any toxicity.

## Results

### PAM induces cancer cell death by increasing intracellular reactive oxidative stress

**Fig. 1:**
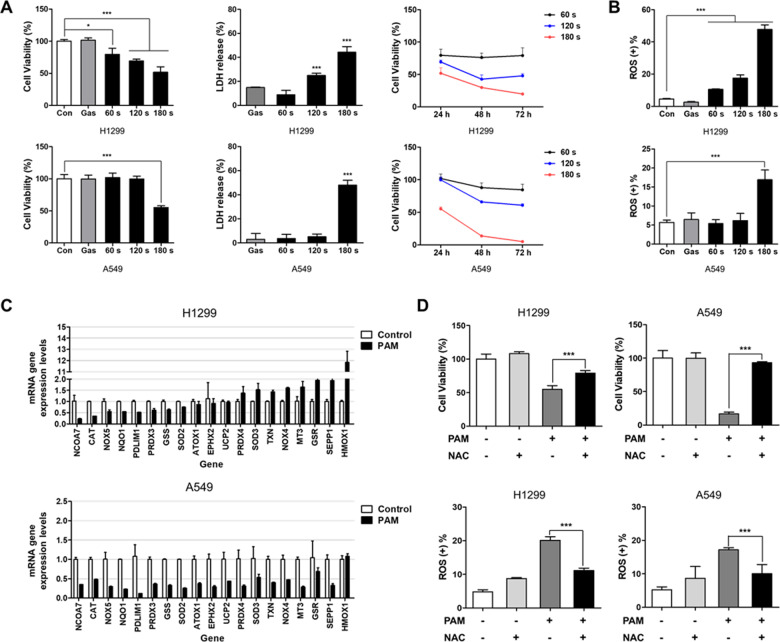
Plasma-activated medium induces cancer cell death by regulating intracellular ROS. The plasma exposure conditions were 1.9 standard liter per minute (SLM), 7 W input power and three exposure times (60, 120, and 180 sec). **A** The cell viability and cytotoxicity were measured using MTT and LDH assay. **B** Intracellular ROS was detected at 24 h after PAM treatment. **C** PAM regulated mRNA expression levels of oxidative stress genes in H1299 and A549 cancer cells (exposure time: 180 sec). **D** N-acetylcysteine (NAC, 2 mM) recovered cell viability and suppressed intracellular ROS by PAM treatment. Cells were treated with PAM (180 sec) for 24 h. Data represent the mean ± SD from three independent experiments (*p < 0.05, **p < 0.01 and ***p < 0.001, as determined by *t* test).

To test whether PAM can induce cell death in human lung cancer cells, H1299 and A549 cells were treated with PAM prepared at different plasma exposure times. The viability of these cancer cells was measured using the MTT assay, and the cytotoxicity was measured using the lactate dehydrogenase (LDH) assay. PAM reduced the viability and increased and the cytotoxicity. In addition, cell viability was further reduced when PAM was used for > 24 h (Fig. 1A). The intracellular ROS level was increased in the PAM-treated H1299 and A549 cells (Fig. 1B). Using qRT-PCR, we investigated whether oxidative stress-related genes changed with PAM treatment (Fig. 1C). The result revealed a PAM-induced altered expression of oxidative stress-related genes. In both cell lines, the gene expression of NCOA7 decreased by < 0.5-fold (Supplementary Table 1). We used ROS scavengers to investigate whether ROS generated by PAM induce cell death. The results showed that N-acetylcysteine (NAC), an ROS scavenging agent, significantly restored cell viability in PAM-treated cells to a level similar to that of control. Moreover, when we co-treated the cells with NAC and PAM, intracellular ROS levels significantly decreased in both cancer cell lines (Fig. 1D). These results indicated that PAM induces cancer cell death by regulating intracellular ROS.

### PAM-induced cancer cell death is associated with increased mitochondrial dysfunction

**Fig. 2:**
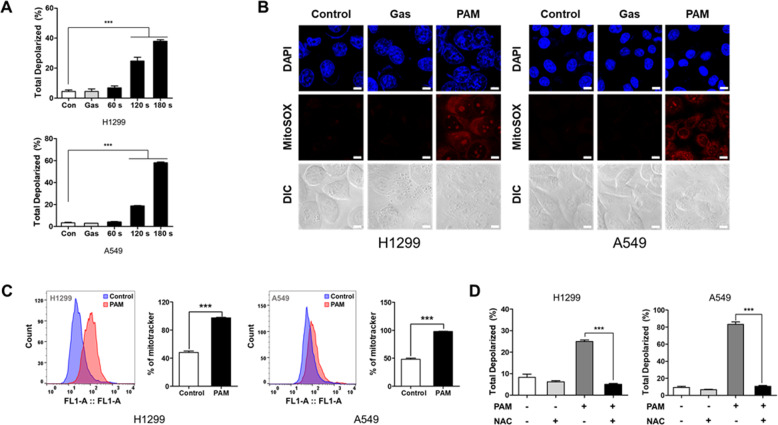
Mitochondrial dysfunction causes by plasma-activated medium. The plasma exposure conditions were 1.9 standard liter per minute (SLM), 7 W input power and three exposure times (60, 120, and 180 sec). **A** PAM increased the proportion of total depolarized mitochondria. Cells were treated with PAM for 24 h. **B** Levels of mitochondrial superoxide was observed by MitoSOX fluorescence using confocal microscopy. H1299 and A549 cells were treated with PAM (180 sec) for 2 h. Scale bar = 10 μm. **C** Mitochondrial biogenesis was detected by Mitotracker using flow cytometry. Cells were treated with PAM (180 s) for 24 h. **D** N-acetylcysteine (NAC) inhibited mitochondrial depolarization by PAM. Cells were treated with PAM (180 s) for 24 h. Data represent the mean ± SD from three independent experiments (***p < 0.001, as determined by *t* test).

ROS can induce cancer cell death by impairing mitochondrial function [41]. To confirm whether PAM affects the mitochondrial function, the mitochondrial membrane potential was measured using the Muse Cell Analyzer. The result indicated that the ratio of depolarized mitochondria was increased in proportion with the plasma exposure time (Fig. 2A). A previous study has reported that ROS can regulate mitochondrial ROS and mass [42, 43]. To validate that ROS generated by PAM leads to increased mitochondrial superoxide levels and biogenesis, we evaluated mitochondrial superoxide level and mass. PAM-induced ROS was found to elevate mitochondrial superoxide level (Fig. 2B). Thus, mitochondrial biogenesis increased significantly upon PAM treatment (Fig. 2C). Interestingly, PAM-induced mitochondrial membrane potential was reversed when NAC was part of the treatment (Fig. 2D). These findings suggest that PAM causes depolarization by upregulating biogenesis and increasing mitochondrial superoxide levels.

### PAM induces ferroptosis in lung cancer cells

**Fig. 3:**
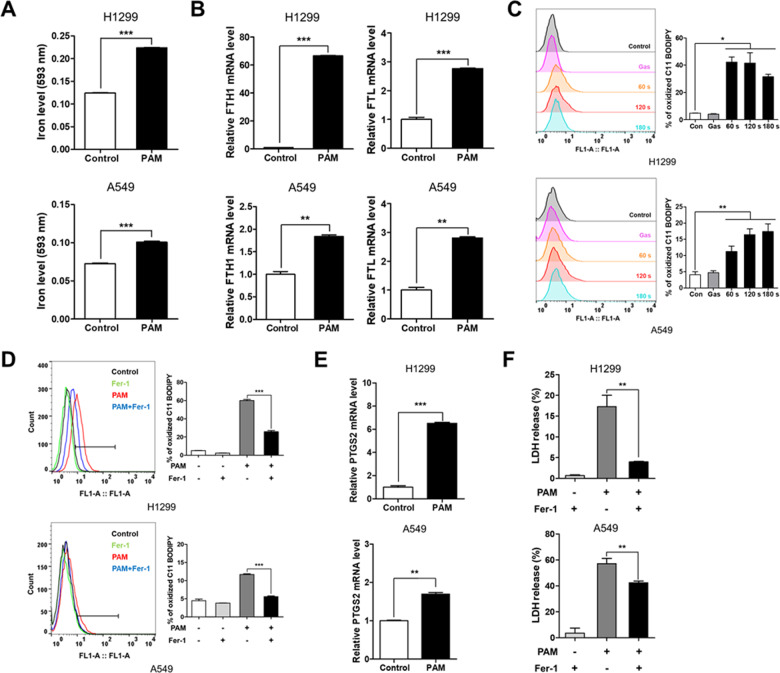
Plasma-activated medium induces ferroptosis in human lung cancer cells. The plasma exposure conditions were 1.9 standard liter per minute (SLM), 7 W input power and three exposure times (60, 120, and 180 s). **A** The expression levels of labile iron were measured using iron assay kit. Indicated cells were treated with PAM (180 s) for 24 h. **B** mRNA expression of FTH1 and FTL was measured by qRT-PCR. **C** PAM-induced lipid ROS levels as assessed by flow cytometry using BODIPY 581/591 C11 dye. H1299 and A549 cells were treated with PAM (180 s) for 2 h. **D** Lipid ROS levels were detected using BODIPY 581/591 C11 dye. The indicated cells were treated PAM (180 s) or 2 μM ferrostatin-1 for 2 h. **E** The expression of PTGS2, the representative ferroptosis marker, was measured by qRT-PCR. **F** H1299 and A549 cells were treated with PAM (180 s) or 2 μM ferrostatin-1 for 24 h. Release of LDH levels was measured using cytotoxicity kit. Data represent the mean ± SD from three independent experiments (*p < 0.05, **p < 0.01 and ***p < 0.001, as determined by *t* test).

Recently, ferroptosis has been identified as a type of mitochondria-related cell death driven by elevated ROS levels [44]. We showed that PAM can induce mitochondrial dysfunction, resulting from increased mitochondrial ROS levels. Thus, we hypothesized that PAM induces ferroptosis. To test this hypothesis, we identified hallmarks of ferroptosis. First, labile iron level was measured using an iron assay. The level of labile iron was observed to increase in both cancer cell lines (Fig. 3A). Increased expression of FTH1 and FTL resulted from upregulated antioxidant defense [45] (Fig. 3B). Second, the accumulation of lipid peroxidation was measured using BODIPY 581/591 C11 staining. The result indicated that the levels of lipid peroxidation were drastically increased in H1299 and A549 cells (Fig. 3C). On the other hand, the level of lipid peroxidation decreased when treated with ferrostatin-1, a ferroptosis inhibitor (Fig. 3D). In accordance with these results, PTGS2, whose levels increase upon ferroptotic stimuli, was upregulated in both H1299 and A549 cells (Fig. 3E). Finally, we confirmed that cytotoxicity was restored by ferrostatin-1, which suggests that ferroptosis is responsible for PAM-induced cell death (Fig. 3F and Fig. S1). These results show that PAM not only promotes ferroptosis but also partially inhibits cell proliferation.

### PAM induces ferroptosis by regulating the NRF2/FSP1 pathway

**Fig. 4:**
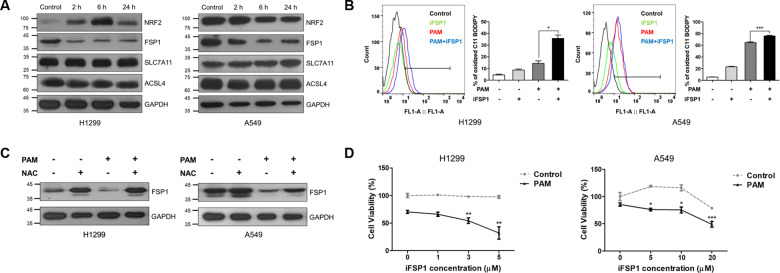
Plasma-activated medium regulates ferroptosis via NRF2/FSP1 signaling. The plasma exposure conditions were 1.9 standard liter per minute (SLM), 7 W input power and 180 s. **A** Western blot analyses of expression of NRF2, FSP1, SLC7A11, and ACSL4 were detected by Western blotting. GAPDH was used as a loading control. **B** Lipid ROS levels were detected using BODIPY 581/591 C11 dye. H1299 and A549 cells were treated with PAM (180 s) or 2 μM ferrostatin-1 for 2 h. **C** The protein levels of FSP1 in H1299 and A549 cells following PAM (180 s) or 2 mM NAC treatments. GAPDH was used as a loading control. **D** The cell viability was measured using MTT assay. H1299 and A549 cells were treated with indicated concentration of FSP1 inhibitor (iFSP1) with or without PAM (180 s). Data represent the mean ± SD from three independent experiments (*p < 0.05, **p < 0.01 and ***p < 0.001, as determined by *t* test).

Subsequently, we sought to elucidate the mechanism though which PAM induces ferroptosis. We confirmed the expression levels of several key proteins involved in ferroptosis pathway and found that both NRF2 and FSP1 protein levels are largely affected by PAM (Fig. 4A). It was found that the expression of NRF2, a well-known suppressor of ferroptosis, increased in response to PAM treatment in H1299 cells, whereas its expression decreased continuously with treatment time in A549 cells. Notably, FSP1, another key ferroptosis suppressor protein, was downregulated in both cancer cell lines treated with PAM. These results suggest that PAM-mediated reduction in FSP1 protein levels contributes to ferroptosis. However, the level of other ferroptosis-related proteins, such GPX4, SLC7A11, and ACSL4, remained unchanged upon PAM treatment.

To investigate the potential role of FSP1 in PAM-induced ferroptosis, we used an FSP1 inhibitor, iFSP1. When iFSP1 was used, PAM-induced lipid peroxidation increased significantly (Fig. 4B). FSP1 inhibition in lung cancer cells leads to the upregulation of lipid ROS. Of note, when both cancer cell lines were co-treated with PAM and NAC, the expression of FSP1 was restored. The level of FSP1 protein increased as the effect of PAM was limited by NAC treatment (Fig. 4C). As expected, the viability of cells treated with in iFSP1 and PAM significantly decreased compared with that of those treated with only PAM (Fig. 4D). Taken together, PAM-generated ROS induce ferroptosis by regulating the expression of FSP1.

### Ferroptosis is involved in PAM-induced tumor suppression in vivo

**Fig. 5:**
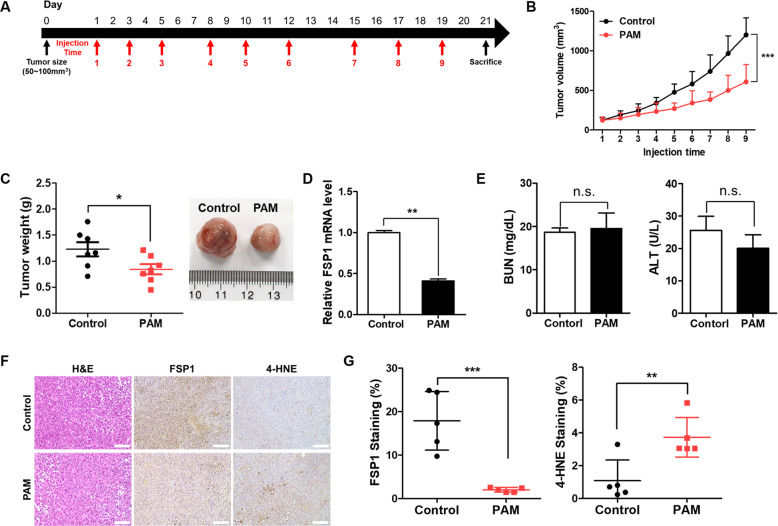
Plasma-activated medium suppresses tumor growth by regulating ferroptosis. The plasma exposure conditions were 1.9 standard liter per minute (SLM), 7 W input power and 180 sec. **A** Mouse treatment schedule. **B** Tumor volumes were determined at injection time points before PAM treatment. Data represent the mean ± SD from seven independent experiments (***p < 0.001, as determined by *t* test). **C** Tumor weights were measured after the tumors were dissected. Data represent the mean ± SD from seven independent experiments (*p < 0.01, as determined by *t* test). **D** The mRNA levels of FSP1 were measured by qRT-PCR. Data represent the mean ± SD from three independent experiments (*p < 0.05, **p < 0.01 and ***p < 0.001, as determined by *t* test). **E** BUN and ALT were analyzed and compared between the control and PAM treatment groups. Data represent the mean ± SD from five independent experiments (n.s.; not significant, as determined by *t* test). **F** Tumor tissue sections were stained with hematoxylin-eosin (H&E) and immunohistochemical staining (FSP1 and 4-HNE). Representative images of stained tumor tissue. Scale bar = 100 μm. **G** Immunochemistry scoring of FSP1 and 4-HNE staining. Data represent the mean ± SD from five independent experiments (**p < 0.01 and ***p < 0.001, as determined by *t* test).

Next we tested whether PAM can induce ferroptosis in vivo. The nude mice implanted with H1299 cells were treated with PAM for a desired period. When tumors reached 100 mm^3^, PAM was injected nine times into the mice [46, 47] (Fig. 5A). Compared with the control group, tumor growth in the PAM-treated group was modestly suppressed by PAM treatment (Fig. 5B). In addition, tumor weight was determined to be lower in the PAM-treated group (Fig. 5C). After isolating the tissue, we analyzed the expression of FSP1 at the mRNA level. As shown in Fig. 5D, the expression of FSP1 decreased dramatically. For liver and kidney toxicity measurements, we analyzed the concentration of alanine aminotransferase (ALT) and blood urea nitrogen (BUN). However, no significant changes in health values were observed (Fig. 5E). Moreover, it was observed that PAM reduced the staining ratio of FSP1 in tumor sample. In contrast, the staining ratio of 4-hydroxynonenal (4-HNE), a lipid peroxidation marker [48], increased (Fig. 5F–G). The expression of PTGS2, which is used as an in vivo indicator of ferroptosis, was also increased (Fig. S2). Collectively, our data strongly suggest that PAM can suppress tumor growth through ferroptosis.

## Discussion

In our previous study, we showed that CAP can generate various reactive oxygen and nitrogen species in the culture medium. CAP-generated PAM increased the levels of intracellular ROS and oxidative stress to ultimately induce apoptosis. We suggest that hydrogen in particular is an important mediator that induces cancer cell death. In this study, we determined the changes in the expression of representative antioxidant genes (Fig. 1C). The lowest expression of NCO7A was noted after PAM treatment. NCOA7 can detect oxidative stress and regulate cellular responses to DNA damage due to oxidative stress. Furthermore, the high expression of NCOA7 in oral squamous cell carcinoma has been suggested as a potential biomarker of the disease [49]. The expression of most antioxidant genes, such as GSS, CAT, SOD2/4, NOX4/6, and NQO1 are generally reduced upon PAM treatment, suggesting that failure in the antioxidant defense mechanism promotes or facilitates PAM-induced ROS production and cell death.

Studies have been frequently conducted on the association between PAM and apoptosis [50–52]. We assumed that PAM may have other anticancer effects and thus explored new pathways associated to ROS. The association between ferroptosis, which was newly proposed in 2012, and PAM has not yet been identified at the molecular level [31]. In this study, we showed that ferroptosis can also be induced by PAM, a newly-studied cancer therapy. After treating lung cancer cells with PAM, we identified several characteristics representing ferroptosis. PAM-induced ROS promoted labile iron level, mitochondrial superoxide level, and lipid peroxidation. Moreover, cell cytotoxicity was decreased upon PAM treatment with a ferroptosis inhibitor (Fig. 3F). This result shows that co-treatment of PAM and inhibitor can synergistically decrease cell viability. Figure 4A shows that the expression of FSP1 was suppressed in both cancer cell lines. Anti-ferroptosis induced by FSP1 is not associated with the expression of GSH, GPX4, or ACSL4 [39]. This was in agreement with our western blotting results. In this study, we did not observe any distinct changes in GPX4 or ACSL4 expression upon PAM treatment. Furthermore, the presence or absence of p53 had no effect on FSP1 expression. Therefore, FSP1 levels could be decreased in both H1299 cell lines without p53 or A549 and with p53.

Interestingly, the change in the expression of NRF2 exhibited an opposite trend in both cells. Presumably, A549 cells harbor a Keap1 mutation, thus, expressing high levels of NRF2 proteins as well as its target genes, thereby protecting cells from oxidative stress such as ferroptotic stimuli. Indeed, basic levels of NRF2 and FSP1 proteins were much higher in A549 cells than in H1299 cells (Fig. 4A). In H1299 cells, NRF2 might be induced by oxidative stress as a negative feedback loop to restore ROS-induced cell damage against PAM. A previous study reported that NRF2 expression was increased in H1299, a KEAP1 wild type, when treated with an oxidative stress inducer similar to our study findings [53, 54]. In particular, HO-1 levels, a target of NRF2, increased up to 12-fold by PAM in H1299 cells, but were consistent in A549 cells (Fig. 1C). HO-1 may promote ferroptosis rather than exerting an antioxidant function, which implies that increased levels of HO-1 contribute toward ferroptosis [55, 56].

**Fig. 6:**
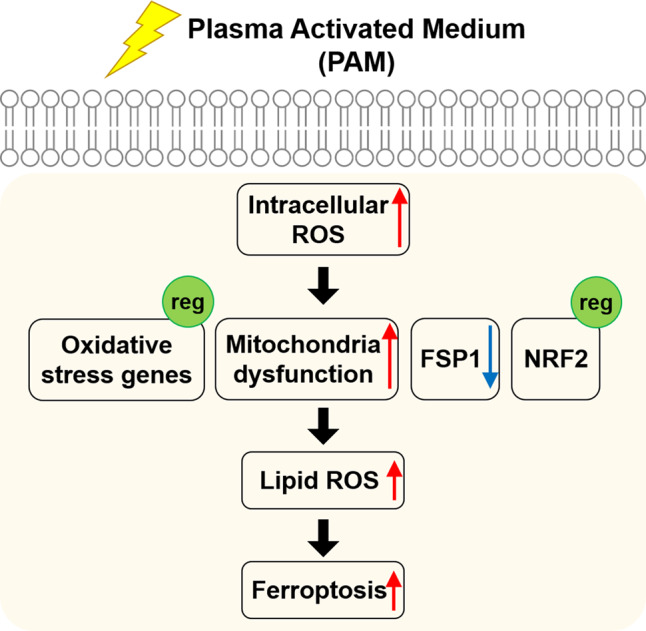
Ferroptosis pathway scheme upon PAM treatment. The schematic description shows the biological impact of PAM on human lung cancer cells. PAM-induced intracellular ROS triggers oxidative stress genes regulation, mitochondria dysfunction, and control of FSP1/NRF2 expression. Subsequently, upregulation of lipid ROS levels ultimately induce ferroptosis.

Our findings show new possibilities for PAM. Over the past few years, most studies have shown that PAM-induced cancer cell death is associated with necrosis, apoptosis, and autophagy. We suggest a novel signaling pathway induced by PAM (Fig. 6). First, PAM can increase the intracellular ROS level. Second, the expression of various oxidative stress genes is regulated. In addition, mitochondrial dysfunction is increased and the expression of FSP1/NRF2 is regulated. Finally, upregulation of lipid ROS ultimately induces ferroptosis. Taken together, our findings suggest that ferroptosis is a new mechanism related to cancer treatment and providing a deeper understanding of PAM in cancer therapy as well.

## Materials and methods

### Cell culture and reagents

Human non-small lung cancer A549 cells, H1299 cells were purchased from American Type Culture Collection (Rockville, MD, USA). A549 cells were grown in Dulbecco Modified Eagle Medium (Capricorn Scientific, Ebsdorfergrund, Germany) supplemented with 10% fetal bovine serum (Capricorn Scientific, Ebsdorfergrund, Germany) and 1% penicillin/streptomycin solution (Capricorn Scientific, Ebsdorfergrund, Germany). H1299 cells were grown in Roswell Park Memorial Institute with l-glutamine media (RPMI, Capricorn Scientific, Ebsdorfergrund, Germany). The cells were maintained in the monolayer at 37 °C in a humidified atmosphere with 5% CO_2_.

Ferrostatin-1 (Cat# S7243), RSL3 (Cat# S8155) were purchased from Selleck Chemicals (Houston, USA). 3-(4, 5-dimethylthiazol-2-yl)-2,5-diphenyltetrazolium bromide (MTT, Cat# M1415) was purchased from Duchefa Biochemie (Harrlem, Netherlands). PrimeScript RT Master mix (Cat# RR036) and TB Green (Cat# RR430) were purchased from TAKARA (Shiga, Japan). The primary antibodies against GAPDH (Cat# 5174) and SLC7A11 (Cat# 12691) were purchased Cell Signaling Technology (Massachusetts, USA). NRF2 (Cat# 16396), FSP1 (Cat# 20886) and ACSL4 (Cat# 22401) were purchased from Proteintech (Rosemont, USA). 4-hydroxynonenal (4-HNE, Cat# ab46546) was purchased from Abcam (Cambridge, UK).

### Plasma sources and preparation of PAM

In producing PAM, a 2.45 GHz commercial microwave-excited atmospheric pressure plasma jet (ME-APPJ) (PM-10, Heuermann HF-Technik GmbH) was used to treat the cell culture medium. The operating conditions of the ME-APPJ included various argon gas flow rates (1.9 SLM) and input powers (7 W). The optical emission spectrum and the gas temperature of ME-APPJ were measured using optical spectrometer (USB-2000+XR-ES Ocean Optics) and a fiber-optic temperature sensor (Luxtron M601-DM&STF), respectively. The cell culture medium was prepared to 3 mL in 60-mm Petri dishes and treated by plasma for 60–180 s. The distance between the open end of quartz tube and surface of cell culture medium was fixed at 5 mm during plasma treatment. And then, the media on top of cells grown in plates was replaced by PAM.

### Cell viability assay

In order to confirm the viability of lung cancer cells, we used MTT solution (5 mg/mL). The cells were seeded in 96-well plates at a density of 7000 cells/well. After the cells are attached, PAM was treated at the desired time. After incubation, PAM was removed and 100 μL MTT solution was added to each well and the cells were further incubated for 3 h. The violet crystals are solubilized with DMSO and the absorbance was measured at 550 nm using microplate reader (FLUO star OPTIMA, BMG Lab tech, Ortenberg, Baden-Wrttemberg, Germany). The relative cell viability (%) was calculated as (O.D. of PAM-treated cells/O.D. of non-treated cells) × 100.

### Cytotoxicity assay

Cytotoxicity was evaluated by measuring the release of LDH in the supernatants from cancer cells cultures with LDH cytotoxicity detection kit (TAKARA, Shiga, Japan) according to the manufacturer’s protocol. At the end of treatment, the cells were treated with reaction mixture for 20 min at room temperature in the dark. The absorbance was measured at 490 nm using a microplate reader. The cytotoxicity (%) was calculated as (O.D. of samples−O.D. of low controls/O.D. of high control−O.D. of low controls) × 100.

### Intracellular ROS detection

The percentage of intracellular ROS was measured using Muse Oxidative Stress Assay Kit (Luminex, Austin, Texas, USA), following the manufacturer’s protocol. In brief, the cells were incubated in 12-well plates (50,000 cells/well) with PAM at 37 °C. At the end of treatment, the cells were washed with 1X PBS buffer and collected in 1.5 mL microtube. Subsequently, the cells were incubated with the Muse Oxidative Stress working solution in the dark at 37 °C for 30 min and quantified using the Muse Cell Analyzer.

### Mitochondria potential detection

Mitochondria potential was confirmed using the mitopotential kit (Luminex, Austin, Texas, USA) according to the manufacturer’s protocol. Briefly, the cells were incubated with the mitopotential working solution for 20 min at 37 °C in a humidified incubator with 5% CO_2_. Subsequently, the 7-AAD reagent added into each samples and incubated for 5 min at room temperature. Each sample was then analyzed using the Muse Cell Analyzer.

### Mitochondrial ROS measurements

To quantify mitochondrial superoxide, we used MitoSOX (Invitrogen, Carlsbad, USA). After the cells were treated with PAM, it was incubated with MitoSOX (500 nM) at 37 °C for 20 min. Data are analyzed using confocal microscope.

### Mitochondrial mass measurements

To detect mitochondrial mass, we used MitoTracker^TM^ Green FM (Thermo fisher scientific, Massachusetts, USA). After cells were treated with PAM, cancer cells were incubated with MitoTracker (40 nM in 0.5 mL 1X PBS) for 15 min. Then, cells were harvested and were added 0.5 mL FACS buffer (1% BSA in 1X PBS). Mitochondrial mass levels were detected using S3e Cell Sorter (Bio-Rad, Hercules, California, USA).

### Labile iron assay

The labile iron concentration was measured using the Iron Assay Kit (Sigma Aldrich, Burlington, USA). Briefly, cells were harvested and homogenized in 5X volumes of Iron Assay Buffer. After getting the supernatant, 5 μL of Iron Reducer was added to each of the sample wells to reduce Fe^3+^ to Fe^2+^ and incubated for 30 min. Subsequently, 100 μL of Iron Probe to each well and incubated the reaction for 60 min. The absorbance was detected at 593 nm using microplate reader.

### Lipid peroxidation assay

For detecting the lipid ROS levels, the cells were seeded on 12-well plates and incubated for attachment. After removing the culture medium, PAM or chemicals were added followed by further incubation of 2 h. Subsequently, cells were treated with 2.5 μM BODIPY 581/591 C11 dye (Invitrogen, Carlsbad, USA) for 15 min in a humidified incubator. Then, the cells were washed with 1X PBS and trypsinized to obtain a single-cell suspension. Lipid peroxidation levels were detected using S3e Cell Sorter (Bio-Rad, Hercules, California, USA).

### Quantitative real-time polymerase chain reaction (qRT-PCR) analysis

Total RNA was extracted with AccuPrep Universal RNA Extraction kit (Bioneer, Daejeon, Korea), and cDNA was synthesized with PrimeScript RT Master mix. Then, quantitative real-time PCR was carried out using TB Green and QuantStudio 3 (Thermo fisher scientific, Massachusetts, USA). The primers were purchased from Bioneer (Accutarget qPCR Screening kit, Bioneer, Daejeon, Korea) (Supplementary Table 2).

### Western blot analysis

Cells were harvested and lysed in RIPA buffer (iNtRON, Seongnam, Korea) containing protease inhibitor and phosphatase inhibitor. Next, the transfer process was carried out using PVDF membrane (Millipore, Billerica, MA, USA). The membranes were blocked with 5% skim-milk (LPS solution, Daejeon, Korea) in 1X TBS-T (1% Tween-20 in Tris-HCl buffer) for 1 h. Subsequently, the membranes incubated with primary antibodies at 4 °C overnight. The secondary antibodies were incubated at room temperature for 1 h. Finally, we were detected using ECL reagent (Dongin, Seoul, Korea) onto an X-ray film (Fuji Film, Tokyo, Japan).

### Mouse

Male 6- to 8-week-old athymic nude mice were purchased from the Slc Inc (Shizuoka, Japan). H1299 cells were collected in cold 1X PBS, and 1 × 10^7^ cells were injected into mice subcutaneously. When the tumor volume reached 100 mm^3^, mice were divided randomly into two groups. Tumors were treated with PAM 3 times a week until a total of 21 treatments were reached. Tumor volume was calculated as volume = length × width^2^ × 1/2. All animal experiments were performed in accordance with protocols approved by the Institutional Animal Care and Use Committee of the Kosin University.

### Hematoxylin & Eosin staining

Tumor tissues were collected and fixed immediately with formalin overnight. Then tissues embedded in paraffin, and cut to a thickness of 5 μm sections. For hematoxylin-eosin (H&E) staining, tissue sections were embedded in xylene for 5 min. Next, tissue sections were sequentially immersed in 100, 90, and 70% ethanol. After exposing the harris hematoxylin for 1 min, the sections were washed with tap water. Then, the sections immersed in eosin for 30 s and it also was washed with tap water. Finally, tissue sections were sequentially immersed in 70, 90, and 100% ethanol for fixing.

### Immunohistochemistry

For immunohistochemistry, the primary antibody used 4-HNE (1:200, Abcam, ab46545), FSP1 (1:200, ProteinTech, 20886), and PTGS2 (1:200, Cell signaling, 12282). In brief, the serial sections were de-paraffinized with xylene and ethanol. Samples were immersed in citrate buffer (pH 6.0), and incubated with boiled water for 20 min. Then the sections were incubated with blocking buffer (2% BSA in 1X PBS) for 10 min at room temperature. The serial sections were incubated with primary antibody for overnight at 4 °C. The antibodies were detected using Peroxidase/DAB kit (DAKO Corporation, Carpinteria, CA, USA). Following fixation, the tissue sections were observed at 200x magnification. Positive staining (%) was calculated using ImageJ software. The staining ratio (%) was calculated as (Positive area / Total area) × 100.

### Statistical analyses

Each experiment was repeated at least three times in all cases. Data are presented as means ± SD (standard deviation). Statistical differences among groups were analyzed using GraphPad Prism 5 (GraphPad Software). In all of the comparisons, a level of *p* < 0.05 was considered significant (n.s., not significant; *p < 0.05; **p < 0.01; ***p < 0.001).

## Supplementary information

Supplementary information

Reproducibility checklist

## Data Availability

Most of the data can be found in the manuscript, and further data used to support the results of this study may also be requested from the corresponding authors.

## References

[1] Fridman G, Friedman G, Gutsol A, Shekhter AB, Vasilets VN, Fridman A (2008). Applied plasma medicine. Plasma Process Polym.

[2] Laroussi M (2009). Low-temperature plasmas for medicine?. IEEE Trans Plasma Sci.

[3] von Woedtke T, Reuter S, Masur K, Weltmann KD (2013). Plasmas for medicine. Phys Rep.

[4] Graves DB. The emerging role of reactive oxygen and nitrogen species in redox biology and some implications for plasma applications to medicine and biology. J Phys D Appl Phys. 2012;45. 10.1088/0022-3727/45/26/263001.

[5] Bekeschus S, Eisenmann S, Sagwal SK, Bodnar Y, Moritz J, Poschkamp B (2020). xCT (SLC7A11) expression confers intrinsic resistance to physical plasma treatment in tumor cells. Redox Biol.

[6] Ishaq M, Evans M, Ostrikov K (2014). Effect of atmospheric gas plasmas on cancer cell signaling. Int J Cancer.

[7] Kang KA, Piao MJ, Eom S, Yoon SY, Ryu S, Kim SB (2020). Non-thermal dielectric-barrier discharge plasma induces reactive oxygen species by epigenetically modifying the expression of NADPH oxidase family genes in keratinocytes. Redox Biol.

[8] Conway GE, Casey A, Milosavljevic V, Liu Y, Howe O, Cullen PJ (2016). Non-thermal atmospheric plasma induces ROS-independent cell death in U373MG glioma cells and augments the cytotoxicity of temozolomide. Br J Cancer.

[9] Kieft IE, Kurdi M, Stoffels E (2006). Reattachment and apoptosis after plasma-needle treatment of cultured cells. IEEE Trans Plasma Sci.

[10] Fridman G, Shereshevsky A, Jost MM, Brooks AD, Fridman A, Gutsol A (2007). Floating electrode dielectric barrier discharge plasma in air promoting apoptotic behavior in Melanoma skin cancer cell lines. Plasma Chem Plasma Process.

[11] Joh HM, Kim SJ, Chung TH, Leem SH. Reactive oxygen species-related plasma effects on the apoptosis of human bladder cancer cells in atmospheric pressure pulsed plasma jets. Appl Phys Lett. 2012;101. 10.1063/1.4742742.

[12] Vandamme M, Robert E, Lerondel S, Sarron V, Ries D, Dozias S (2012). ROS implication in a new antitumor strategy based on non-thermal plasma. Int J Cancer.

[13] Keidar M, Shashurin A, Volotskova O, Ann Stepp M, Srinivasan P, Sandler A, et al. Cold atmospheric plasma in cancer therapy. Phys Plasmas. 2013;20. 10.1063/1.4801516.

[14] Schmidt A, von Woedtke T, Vollmar B, Hasse S, Bekeschus S (2019). Nrf2 signaling and inflammation are key events in physical plasma-spurred wound healing. Theranostics.

[15] Adachi T, Tanaka H, Nonomura S, Hara H, Kondo SI, Hori M (2015). Plasma-activated medium induces A549 cell injury via a spiral apoptotic cascade involving the mitochondrial-nuclear network. Free Radic Biol Med.

[16] Bauer G (2019). Intercellular singlet oxygen-mediated bystander signaling triggered by long-lived species of cold atmospheric plasma and plasma-activated medium. Redox Biol.

[17] Azzariti A, Iacobazzi RM, Di Fonte R, Porcelli L, Gristina R, Favia P (2019). Plasma-activated medium triggers cell death and the presentation of immune activating danger signals in melanoma and pancreatic cancer cells. Sci Rep.

[18] Nguyen NH, Park HJ, Hwang SY, Lee JS, Yang SS (2019). Anticancer efficacy of long-term stored plasma-activated medium. Appl Sci.

[19] Bauer G, Sersenová D, Graves DB, Machala Z (2019). Cold atmospheric plasma and plasma-activated medium trigger RONS-based tumor cell apoptosis. Sci Rep.

[20] Sagwal SK, Pasqual-Melo G, Bodnar Y, Gandhirajan RK, Bekeschus S. Combination of chemotherapy and physical plasma elicits melanoma cell death via upregulation of SLC22A16. Cell Death Dis. 2018;9. 10.1038/s41419-018-1221-6. 10.1038/s41419-018-1221-6 PMC6281583 30518936

[21] Lee J, Han SJ, Kang HY, Wi S, Jung M. On–off switching of cell cycle and melanogenesis regulation of melanocytes by non-thermal atmospheric pressure plasma-activated medium. Sci Rep. 2019;9:13400. 10.1038/s41598-019-50041-2 PMC6746696 31527659

[22] Yang X, Chen G, Yu KN, Yang M, Peng S, Ma J, et al. Cold atmospheric plasma induces GSDME-dependent pyroptotic signaling pathway via ROS generation in tumor cells. Cell Death Dis. 2020;11. 10.1038/s41419-020-2459-3. 10.1038/s41419-020-2459-3 PMC7186223 32341339

[23] Malyavko A, Yan D, Wang Q, Klein AL, Patel KC, Sherman JH (2020). Cold atmospheric plasma cancer treatment, direct versus indirect approaches. Mater Adv.

[24] Mohamed H, Gebski E, Reyes R, Beane S, Wigdahl B, Krebs FC (2021). Differential effect of non-thermal plasma RONS on two human leukemic cell populations. Cancers (Basel).

[25] Joh HM, Choi JY, Kim SJ, Chung TH, Kang TH (2014). Effect of additive oxygen gas on cellular response of lung cancer cells induced by atmospheric pressure helium plasma jet. Sci Rep.

[26] Kim DY, Kim SJ, Joh HM, Chung TH. Characterization of an atmospheric pressure plasma jet array and its application to cancer cell treatment using plasma activated medium. Phys Plasmas. 2018;25. 10.1063/1.5037249.

[27] Jo A, Joh HM, Chung JW, Chung TH (2020). Cell viability and measurement of reactive species in gas- and liquid-phase exposed by a microwave-excited atmospheric pressure argon plasma jet. Curr Appl Phys.

[28] Jo A, Joh HM, Chung TH, Chung JW (2020). Anticancer effects of plasma-activated medium produced by a microwave-excited atmospheric pressure argon plasma jet. Oxid Med Cell Longev.

[29] Turrini E, Laurita R, Simoncelli E, Stancampiano A, Catanzaro E, Calcabrini C (2020). Plasma-activated medium as an innovative anticancer strategy: Insight into its cellular and molecular impact on in vitro leukemia cells. Plasma Process Polym.

[30] Yoshikawa N, Liu W, Nakamura K, Yoshida K, Ikeda Y, Tanaka H (2020). Plasma-activated medium promotes autophagic cell death along with alteration of the mTOR pathway. Sci Rep.

[31] Dixon SJ, Lemberg KM, Lamprecht MR, Skouta R, Zaitsev EM, Gleason CE (2012). Ferroptosis: an iron-dependent form of nonapoptotic cell death. Cell.

[32] Lei G, Zhang Y, Koppula P, Liu X, Zhang J, Lin SH (2020). The role of ferroptosis in ionizing radiation-induced cell death and tumor suppression. Cell Res.

[33] Kerins MJ, Ooi A (2018). The roles of NRF2 in modulating cellular iron homeostasis. Antioxid Redox Signal.

[34] Park TJ, Park JH, Lee GS, Lee JY, Shin JH, Kim MW, et al. Quantitative proteomic analyses reveal that GPX4 downregulation during myocardial infarction contributes to ferroptosis in cardiomyocytes. Cell Death Dis. 2019;10. 10.1038/s41419-019-2061-8. 10.1038/s41419-019-2061-8 PMC6828761 31685805

[35] Yang WS, Sriramaratnam R, Welsch ME, Shimada K, Skouta R, Viswanathan VS (2014). Regulation of ferroptotic cancer cell death by GPX4. Cell.

[36] Kim J, Keum YS (2016). NRF2, a key regulator of antioxidants with two faces towards cancer. Oxid Med Cell Longev.

[37] He F, Ru X, Wen T (2020). NRF2, a transcription factor for stress response and beyond. Int J Mol Sci.

[38] Dodson M, Castro-Portuguez R, Zhang DD (2019). NRF2 plays a critical role in mitigating lipid peroxidation and ferroptosis. Redox Biol.

[39] Doll S, Freitas FP, Shah R, Aldrovandi M, da Silva MC, Ingold I (2019). FSP1 is a glutathione-independent ferroptosis suppressor. Nature.

[40] Bersuker K, Hendricks JM, Li Z, Magtanong L, Ford B, Tang PH (2019). The CoQ oxidoreductase FSP1 acts parallel to GPX4 to inhibit ferroptosis. Nature.

[41] Perillo B, Di Donato M, Pezone A, Di Zazzo E, Giovannelli P, Galasso G (2020). ROS in cancer therapy: the bright side of the moon. Exp Mol Med.

[42] Yadav N, Kumar S, Marlowe T, Chaudhary AK, Kumar R, Wang J, et al. Oxidative phosphorylation-dependent regulation of cancer cell apoptosis in response to anticancer agents. Cell Death Dis. 2015;**6**. 10.1038/cddis.2015.305. 10.1038/cddis.2015.305 PMC4670921 26539916

[43] Shin HJ, Kwon HK, Lee JH, Anwar MA, Choi S (2016). Etoposide induced cytotoxicity mediated by ROS and ERK in human kidney proximal tubule cells. Sci Rep.

[44] Gao M, Yi J, Zhu J, Minikes AM, Monian P, Thompson CB (2019). Role of mitochondria in ferroptosis. Mol Cell.

[45] Gao M, Monian P, Pan Q, Zhang W, Xiang J, Jiang X (2016). Ferroptosis is an autophagic cell death process. Cell Res.

[46] Adhikari M, Adhikari B, Kaushik N, Lee SJ, Kaushik NK, Choi EH. Melanoma growth analysis in blood serum and tissue using xenograft model with response to cold atmospheric plasma activated medium. Appl Sci. 2019;9. 10.3390/app9204227.

[47] Li W, Yu H, Ding D, Chen Z, Wang Y, Wang S (2019). Cold atmospheric plasma and iron oxide-based magnetic nanoparticles for synergetic lung cancer therapy. Free Radic Biol Med.

[48] Yang Y, Luo M, Zhang K, Zhang J, Gao T, Connell DO (2020). Nedd4 ubiquitylates VDAC2/3 to suppress erastin-induced ferroptosis in melanoma. Nat Commun.

[49] Xie X, Jiang Y, Yuan Y, Wang P, Li X, Chen F (2016). MALDI imaging reveals NCOA7 as a potential biomarker in oral squamous cell carcinoma arising from oral submucous fibrosis. Oncotarget.

[50] Bengtson C, Bogaerts A (2020). On the anti-cancer effect of cold atmospheric plasma and the possible role of catalase-dependent apoptotic pathways. Cells.

[51] Jawaid P, Rehman MU, Zhao QL, Misawa M, Ishikawa K, Hori M, et al. Small size gold nanoparticles enhance apoptosis-induced by cold atmospheric plasma via depletion of intracellular GSH and modification of oxidative stress. Cell Death Discov. 2020;6. 10.1038/s41420-020-00314-x. 10.1038/s41420-020-00314-x PMC7483448 32963811

[52] Alizadeh E, Ptasińska S (2021). Recent advances in plasma-based cancer treatments: approaching clinical translation through an intracellular view. Biophysica.

[53] Torrente L, Prieto-Farigua N, Falzone A, Elkins CM, Boothman DA, Haura EB (2020). Inhibition of TXNRD or SOD1 overcomes NRF2-mediated resistance to β-lapachone. Redox Biol.

[54] Baird L, Yamamoto M (2020). The molecular mechanisms regulating the KEAP1-NRF2 pathway. Mol Cell Biol.

[55] Hassannia B, Wiernicki B, Ingold I, Qu F, Van Herck S, Tyurina YY (2018). Nano-targeted induction of dual ferroptotic mechanisms eradicates high-risk neuroblastoma. J Clin Invest.

[56] Kwon MY, Park E, Lee SJ, Chung SW (2015). Heme oxygenase-1 accelerates erastin-induced ferroptotic cell death. Oncotarget.

