# Supplementary material for: Plasma-activated medium induces ferroptosis by depleting FSP1 in human lung cancer cells
Source: Cell Death Dis. 2022 Mar 7;13(3):212. doi: 10.1038/s41419-022-04660-9 (PMC8901787; doi:10.1038/s41419-022-04660-9)
Supplement: Supplementary file 1 — Supplementary information [file 41419_2022_4660_MOESM1_ESM.docx]

**Supplementary materials**

**Supplementary Figure 1**


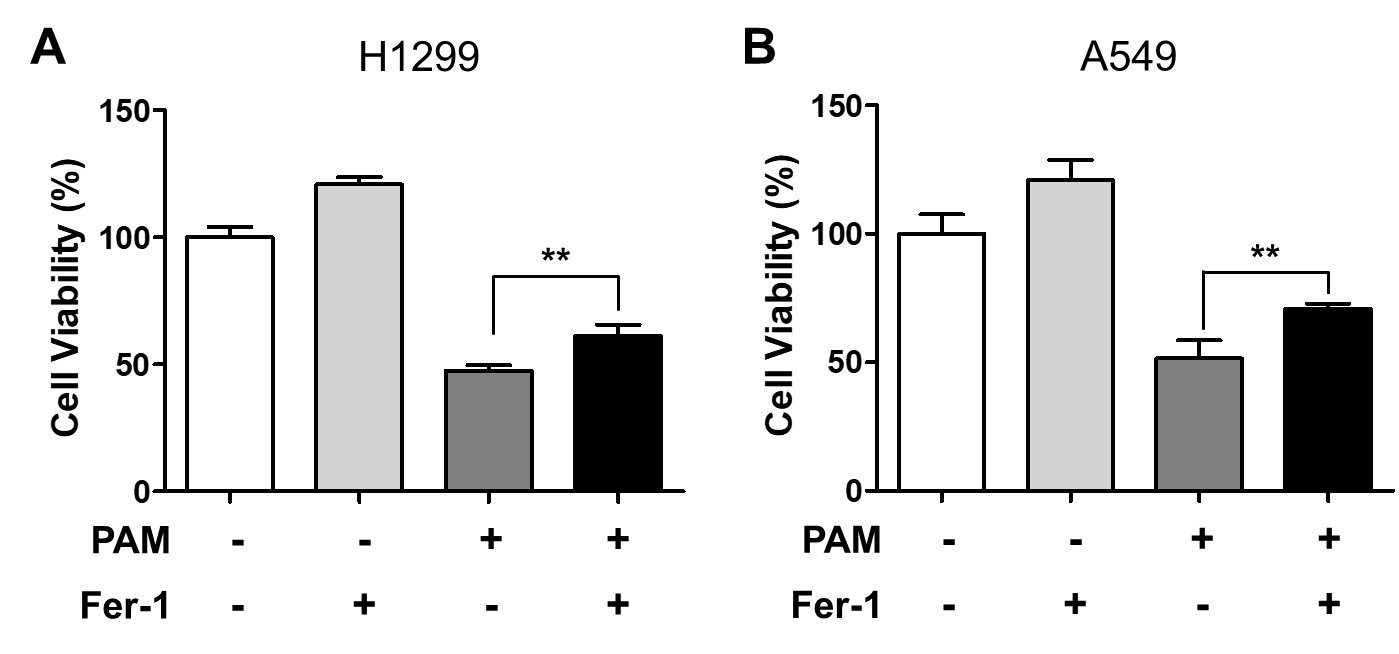


**Fig S1. Ferroptosis inhibitor restores human lung cancer cell death by PAM.** The cell viability and cytotoxicity were measured using MTT assay. Ferrostatin-1(Fer-1, 2 μM) recovered cell viability by PAM (180 sec) on H1299 cells (**A**) and A549 cells (**B**). Data represent the mean ± SD from three independent experiments (***p* < 0.01, as determined by t-test).

**Supplementary Figure 2**


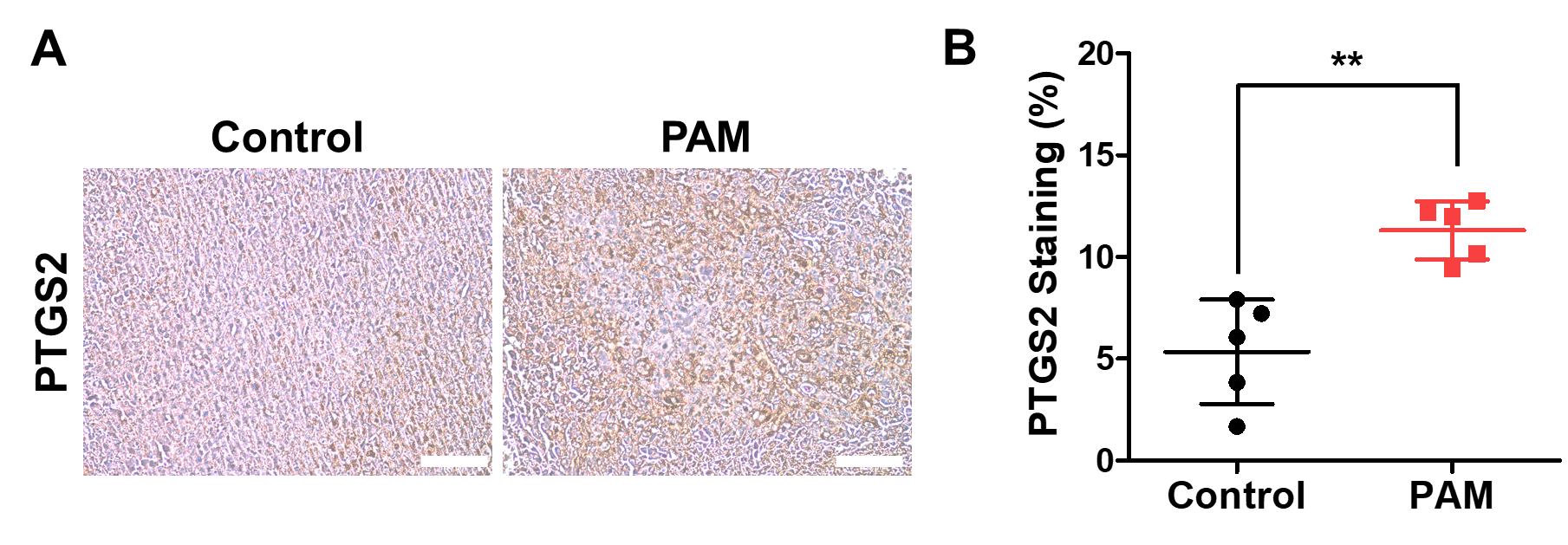


**Fig S2.** **Plasma-activated medium increases the protein level of PTGS2, the representative ferroptosis marker.** (**A**) Tumor tissue sections were stained with immunohistochemical staining. Representative images of stained tumor tissue. Scale bar = 100 μm. (**B**) Immunochemistry scoring of PTGS2 staining. Data represent the mean ± SD from five independent experiments (***p* < 0.01, as determined by t-test).

**Supplementary Table 1**

| ***Target gene*** | ***H1299*** | | ***A549*** | |
| --- | --- | --- | --- | --- |
|  | ***Fold***  ***Change**** | ***Correted***  ***P-value*** | ***Fold***  ***change*** | ***Correted***  ***P-value*** |
| **NCOA7** | 0.2290 | 0.0484 | 0.3500 | 0.0030 |
| **CAT** | 0.3525 | 0.0002 | 0.4795 | 0.0053 |
| **NOX5** | 0.5520 | 0.0431 | 0.2965 | 0.0284 |
| **NQO1** | 0.5450 | 0.0012 | 0.2310 | <0.0001 |
| **PDLIM1** | 0.5205 | 0.0069 | 0.1180 | 0.0444 |
| **PRDX3** | 0.6140 | 0.0209 | 0.3665 | 0.0047 |
| **GSS** | 0.6305 | 0.0218 | 0.3260 | 0.0006 |
| **SOD2** | 0.7485 | 0.0019 | 0.2545 | 0.0002 |
| **ATOX1** | 0.8705 | 0.3600 | 0.3715 | 0.0097 |
| **EPHX2** | 0.9225 | 0.7419 | 0.2900 | 0.0185 |
| **UCP2** | 0.9610 | 0.5791 | 0.4365 | 0.0447 |
| **PRDX4** | 1.3730 | 0.2267 | 0.3110 | 0.0496 |
| **SOD3** | 1.5260 | 0.1200 | 0.5340 | 0.1609 |
| **TXN** | 1.4360 | 0.0154 | 0.4025 | 0.0081 |
| **NOX4** | 1.5995 | 0.0050 | 0.4715 | 0.0189 |
| **MT3** | 1.6470 | 0.1096 | 0.2860 | 0.0037 |
| **GSR** | 1.9530 | 0.0712 | 0.6855 | 0.3695 |
| **SEPP1** | 1.9635 | 0.0095 | 0.3290 | 0.0160 |
| **HMOX1** | 11.8365 | 0.0042 | 1.0790 | 0.4400 |

*Fold changes were calculated by comparison of the mean expression value in the PAM group to the control group.

**Supplementary Table 2**

| ***P number*** | ***Gene name*** | ***Accession number*** | ***Product size*** |
| --- | --- | --- | --- |
| P222440 | NCOA7 | NM_001122842.2 | 148 |
| P185140 | CAT | NM_001752.3 | 149 |
| P210846 | NOX5 | NM_001184779.1 | 148 |
| P113225 | NQO1 | NM_000903.2 | 158 |
| P144216 | PDLIM1 | NM_020992.3 | 150 |
| P302497 | PRDX3 | NM_001302272.1 | 149 |
| P278341 | GSS | NM_000178.3 | 143 |
| P299527 | SOD2 | NM_000636.3 | 158 |
| P260939 | ATOX1 | NM_004045.3 | 149 |
| P120215 | EPHX2 | NM_001256482.1 | 153 |
| P198975 | UCP2 | NM_003355.2 | 150 |
| P266830 | PRDX4 | NM_006406.1 | 139 |
| P290739 | SOD3 | NM_003102.2 | 113 |
| P115235 | TXN | NM_001244938.1 | 149 |
| P176436 | NOX4 | NM_0011443836.2 | 150 |
| P298148 | MT3 | NM_005954.3 | 135 |
| P209025 | GSR | NM_000637.4 | 145 |
| P306058 | SEPP1 | NM_001085486.2 | 153 |
| P133045 | HMOX1 | NM_002133.2 | 167 |
